# Maintenance Therapy Post-Hematopoietic Stem Cell Transplantation in Acute Myeloid Leukemia

**DOI:** 10.3390/curroncol31100451

**Published:** 2024-10-10

**Authors:** Martina Canichella, Matteo Molica, Carla Mazzone, Paolo de Fabritiis

**Affiliations:** 1 Hematology, St. Eugenio Hospital, ASL Roma2, 00144 Rome, Italy; 2 Department of Hematology-Oncology, Azienda Ospedaliera Pugliese-Ciaccio, 88100 Catanzaro, Italy; molica@bce.uniroma1.it; 3 Department of Biomedicina e Prevenzione, Tor Vergata University, 00133 Rome, Italy

**Keywords:** acute myeloid leukemia (AML), hematopoietic stem cell transplantation (HSCT), maintenance therapy post-HSCT in AML, targeted drugs, cellular therapy, hypometylating agents

## Abstract

High-risk acute myeloid leukemia has been associated with a poor outcome. Hematopoietic stem cell transplantation (HSCT) represents the only curative option for eligible patients. Relapse after HSCT is a dramatic event with poor chances of survival. With the aim of reducing the rate of post-HSCT relapse, maintenance treatment has been investigated in this setting. Results from clinical trials suggest an advantage in the use of a maintenance strategy; however, standardized guidelines are not yet available due to the lack of prospective clinical trials. In this review, we have reported the most important strategies adopted as post-HSCT maintenance, highlighting their efficacy, but the current research also opens questions.

## 1. Introduction

Acute myeloid leukemia (AML) is a very heterogeneous group of clonal diseases, which arises from clonal expansion of leukemic blasts in the bone marrow, peripheral blood, and extramedullary sites [1]. The prognosis of AML is influenced by several factors, including the patient’s age and comorbidities, cytogenetic and mutational profiles, and measurable residual disease (MRD) assessment. The latter can be detected with different methods ranging from flow cytometry to molecular methods—real time PCR and more recently NGS—which allow the identification of recurrence at an increasingly deeper level. For high-risk patients, if eligible, hematopoietic stem cell transplantation (HSCT) is a strongly recommended consolidation strategy. In the last decades, despite advances in reducing transplant-related mortality (TRM), the relapse post-HSCT remained high with a median survival of 4–6 months and a 1-year survival rate of less than 20% [2,3,4]. For more than 3 decades, a growing body of research has focused on maintenance post-HSCT as a strategy to prevent relapse. Unfortunately, the results of clinical trials did not lead to conclusive results, leaving several issues unsolved. In this review, we discussed the limitations and the advantages of maintenance therapy post-HSCT, focusing on the three main strategies currently under investigation: targeted therapies, hypomethylating (HMA) agents, and donor lymphocyte infusions (DLI). Each of these therapies offer different mechanisms of action and potential benefits, but they also come with unique challenges and considerations.

## 2. Maintenance Therapy in AML: Is It Time to Refine the Definition?

Compared with B-cell acute lymphoblastic leukemia (B-ALL) with standard class risk and high-risk acute promyelocytic leukemia (APL), in which the maintenance is a well-established and effective phase, in AML, different maintenance trials failed to demonstrate any improvement in survival [5].

By definition, the maintenance phase has been considered as low-intensity therapy administered over a relatively long time after achieving at least complete hematological remission (CHR). The aim of the maintenance phase is to avoid clonal evolution and, at the same time, not to add significant toxicity in terms of infection risk, transfusion support, and reduction in quality of life. Indeed, the ideal drug for the maintenance phase should be easy to administer with a low financial burden. CC-486, an oral formulation of azacitidine (AZA), reflects these features, and it has been approved as maintenance treatment for AML patients in CHR who have not immediately undergone HSCT [6]. The approval of CC-486 by the Food and Drug Administration (FDA) and the European Medicines Agency (EMA) is based on the results of the landmark QUAZAR trial, in which the prerequisite for initiating maintenance was the achievement of CHR regardless of the depth of response. However, despite the initial advantage in overall survival (OS) of the CC-486 arm compared to the placebo, with a longer follow-up, the survival curve did not reach the plateau, suggesting that the maintenance phase in AML still presents many unsolved issues. Firstly, with the widespread application of MRD assessment in AML, the exact cutoff for starting maintenance is unclear. According to MRD monitoring, wo scenarios of treatment are possible: prophylaxis when MRD is negative and the pre-emptive state when MRD remains or becomes positive. In the post-HSCT setting, chimerism monitoring is a useful tool to predict relapse. The optimal zone of activity of maintenance could be between negativity and positivity at low levels, but it is essential to consider the patient’s clinical conditions, the AML risk class, and the intensity of the previous treatment. Another fundamental issue to be clarified is the time point of MRD evaluation to start maintenance. Indeed, in the setting of post-HSCT, maintenance therapy represents a more complex challenge [7]. Immune surveillance post-transplant can be considered a form of maintenance, involving the balance between graft-versus-leukemia (GvL) effects and the risk of graft-versus-host disease (GvHD). The ideal drug for maintenance post-HSCT, in addition to including the features mentioned above, should avoid exacerbating GvHD while still maintaining effective GvL activity. Further clinical studies and, consequently, the consensus of expert panels are needed to define these aspects to drive the initiation of pre- and post-HSCT maintenance.

## 3. Who Are the Candidates for Maintenance Treatment?

Maintenance therapy inevitably exposes patients to additional toxicity. So, it is extremally important to select those cases in which the potential benefits—and toxicity—of maintenance drugs counterbalance the risk of relapse [8]. Factors to consider are the adverse cytogenetic and/or molecular features and the MRD state pre- and post-HSCT. Indeed, in recent years, deep characterization of the AML molecular landscape has revealed those mutations predictive to responses to maintenance therapy. Another important aspect to consider for maintenance is the clinical situation of patients. The last aspect requires careful evaluation in the setting of post-HSCT in which the presence of active GvHD, cytopenia, or organ dysfunction, make the administration of maintenance drugs more complicated. Furthermore, there is limited evidence of drug efficacy and few approved compound options for maintenance phase post-HSCT. A more refined approach with novel drugs or combinations, supported by comprehensive clinical evidence, is essential to optimize maintenance strategies and improve the prognosis for high-risk patients. Below, we illustrate the results of clinical trials of the maintenance phase post-HSCT (Table 1).

## 4. Targeted Drugs

**Figure 1:**
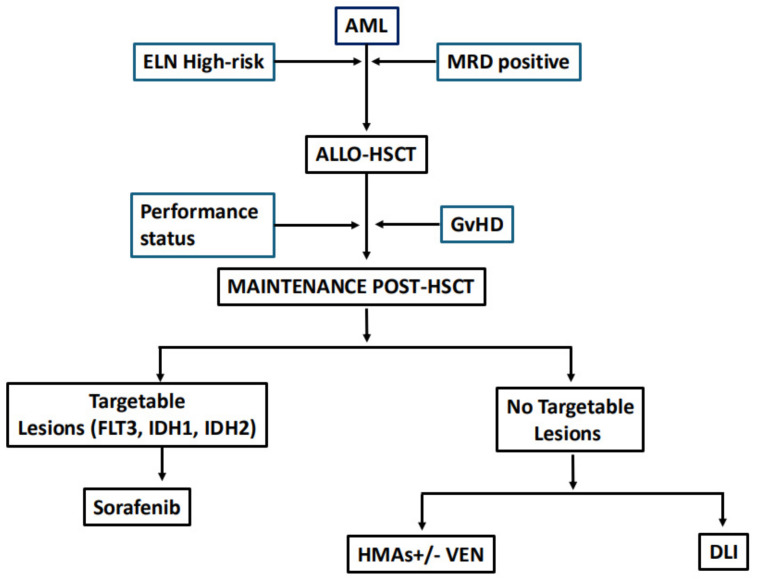
Proposed algorithm for the choice of maintenance therapy. ELN: European Leukemia Network; MRD: measurable residual disease; ALLO-HSCT: allogeneic-stem cell transplantation; GvHD: graft-versus-host disease; HMA: hypomethylating agents; VEN: venetoclax.

The targeted drugs introduced in the treatment of specific AML subtypes attracted attention for their possible use in the maintenance phase post-HSCT. In the next section, we illustrate the experience of the most promising compounds. Targeted drugs could be the primary choice of maintenance for patients harboring specific molecular lesions (Figure 1).

### 4.1. FLT3 Inhibitors (FLT3i)

Fms-like tyrosine kinase 3 (FLT3) is one of the most mutated genes in AML, and it can be hit by two different genetic lesions, the internal tandem duplication (ITD) is the most frequent, occurring in almost 25% of cases, and the missense mutations of tyrosine kinase domain (TKD), recurring in 5–10% of cases [9,10,11,12,13]. Both mutations result in constitutive activation of FLT3 signaling (MAPK and PI3K pathways), leading to the leukemogenesis process. The prognosis of AML with FLT3-ITD is poor with low survival rates [14]. For these reasons, FLT3-ITD and TKD have become the focus of targeted therapy. The FLT3 inhibitors (FLT3i) may be categorized into two types. The first binds the active FLT3 conformation and is effective against both ITD and TKD mutations. The second links the inactive form of FLT3-ITD but not the TKD mutation. In the setting of maintenance post-HSCT, sorafenib and gilteritinib present more robust evidence of effectiveness post-transplant than midostaurin and quizartinib. Crenolanib is currently under investigation in an ongoing clinical trial (NCT02400255). From the first experience in 2015 by Antar et al., which reported the efficacy and safety of sorafenib maintenance in six allografted patients, different retrospective series have confirmed these results [15]. In a preclinical study, sorafenib was demonstrated to induce the production of IL-15 from myeloid blast cells, which activate the CD8+ T cells with a possible GvL effect [16]. Then, the German phase II randomized trial SORMAIN enrolled 83 patients with FLT3-ITD AML who underwent HSCT in the first or in subsequent CR. The maintenance with sorafenib (*n* = 43) or the placebo (*n* = 40) were administered for 2 years at a dosage of 400 mg BID. With a median follow-up of 42 months, the sorafenib arm showed a better 24-month OS compared with the placebo (90% vs. 66%, respectively) without increased GvHD. Indeed, the advantage of sorafenib was evident regardless of MRD status at the time of transplantation. MRD-positive patients in the sorafenib cohort had a statistically significantly better relapse-free survival (RFS) than patients treated with the placebo [17]. Another experience derived from a Chinese study group demonstrated in a randomized phase 3 trial that sorafenib maintenance after HSCT improved OS and reduced relapse compared with patients not allografted. These results were also confirmed in a post-hoc analysis with a 5-year follow-up [18]. Gilteritinib was demonstrated for the first time to be effective post-HSCT in a retrospective experience by the Japan group. Subsequently, Perl et al. published the results of a post-hoc analysis of the phase III ADMIRAL study regarding the survival of patients with FLT3 R/R AML who received post-HSCT gilteritinib as maintenance. The results showed that the patients who resumed gilteritinib had a lower relapse rate and a better OS compared with those who did not receive it. Indeed, the inhibitor was well tolerated [19]. However, the major limitations were the small sample of patients and the absence of a second randomization for the maintenance phase. More recently, gilteritinib was tested post-HSCT by the BMT-CNT group in the randomized phase III MORPHO trial. The trial enrolled 356 patients; gilteritinib was administered at a dosage of 120 mg/day for 24 months post-HSCT. This trial provided the MRD evaluation by NGS with a level of detection of 1 × 10^−6^. The primary end point was RFS. The results showed a higher RFS in the gilteritinib group compared to the placebo without reaching statistical significance. A subanalysis demonstrated that the main benefit of gilteritinib as post-HSCT maintenance was for patients with MRD positivity before starting treatment rather than those with negative MRD [20]. About midostaurin, the effectiveness of which in combination with chemotherapy in first-line treatment has been proven in the phase 3 RATIFY/CALGB 10,603 trial, there are few experiences in the maintenance phase post-HSCT. The same RATIFY did not provide continued midostaurin as maintenance for patients allografted [21]. The phase II randomized RADIUS trial evaluated the midostaurin post-HSCT FLT3-ITD+ AML patients in first complete remission (CR1) but did not reveal significantly important differences. Finally, quizartinib demonstrated promising responses post-HSCT in the QuANTUM-first and subsequently in a phase I trial,; however, the results should be confirmed in a randomized clinical trial with a greater number of patients [22]. Based on this evidence, maintenance therapy in FLT3+ AML is strongly recommended in patients with persistently positive MRD post-HSCT. The duration of the maintenance phase is commonly accepted to be 24 months. However, further studies may identify additional molecular characteristics which will help to identify those patients who really benefit from a maintenance phase.

### 4.2. IDH Inhibitors

Isocitrate dehydrogenase (IDH)1 and IDH2 genes encode for two enzymes implicated in the catabolism of isocitrate to α-ketoglutarate. IDH1 and IDH2 mutations are recurrent in about 10% of AML [23]. Ivosidenib and enasidenib are two oral drugs which inhibit IDH1 and IDH2 mutated proteins, respectively; these drugs are indicated as monotherapy for R/R IDH1 and IDH2-mutated AML cases. Both drugs have been investigated post-HSCT. In a phase I trial, enasidenib was administered in 19 patients between 30 and 90 days from allotransplant at a dosage of 100 mg daily for 12 cycles. The 2-year progression-free survival (PFS) and OS were 69% and 74%, respectively, while the cumulative incidence of relapse (CIR) was 16%. GvHD events were limited. Similarly, ivosidenib 500 mg was utilized after HSCT in 16 patients as maintenance. Two-year CIR and non-relapse mortality (NRM) were 19% and 0%, respectively. The 2-year PFS and OS were 81% and 88%, respectively. These inhibitors demonstrated promising results and safety profiles with limited toxicity and low risk of GvHD, which led them to be considered as ideal drugs for maintenance post-HSCT in IDH1 and IDH2 positive AML cases. Altogether, despite the small number of patients, these data were favorable compared with the historical experiences [24,25]. However, larger prospective trials are necessary to confirm these results.

### 4.3. Eprenetapopt

Eprenetapopt (APR-246) is a first-in-class, small-molecule p53 re-activator tested in combination with azacitidine in maintenance post-HSCT in *TP53* mutated (TP53m) AML or MDS. Encouraging results derived from phase II clinical trials which enrolled 55 patients with TP53m AML or MDS [26]. A total of 33 of these underwent HSCT and started the maintenance therapy with eprenetapopt (3.7 g once daily intravenously on days 1–4) plus AZA (32 mg/m^2^ once daily intravenously/subcutaneously on days 1–5). The 1-year RFS and OS probability were 60% and 79%, respectively; the median RFS and OS were 12.5 and 20.6 months, respectively. Treatment was well tolerated with no increase in GVHD. These data demonstrated superior results compared with the previous published outcome of TP53m MDS and AML patients who underwent HSCT without the subsequent maintenance phase [27,28]. However, the preliminary results of the A phase III trial (NCT03745716) with eprenetapopt in maintenance post-HSCT did not confirm this effectiveness.

## 5. Hypomethylating Agents (Hma) Alone or in Combination

HMA (azacitidina-AZA- and decitabina-DEC) are nucleoside analogs that irreversibly link the methylating enzymes and lead to cellular apoptosis. These drugs have revolutionized the treatment of unfit AML patients, inducing a sustained response with a good safety profile. AZA has been used post-HSCT both with negative and persistently positive MRD (pre-emptive) [29]. The first report about AZA as maintenance in the post-HSCT setting dates from 2010 by de Lima and colleagues. In a dose-finding design, the researchers found that the optimal schedule of AZA was 32 mg/m^2^ for 5 days in a 30-day cycle. The 1-year OS and event-free survival (EFS) rates were 77% and 58%, respectively [30]. Subsequently, several studies revealed the immunological property of AZA increasing the response of tumor-specific CD8+ T-cells upregulating silenced tumor antigens, HLA, and costimulatory molecules on the leukemic cells [31]. Furthermore, AZA demonstrated to upregulate inhibitory pathways of regulatory T-cells preventing GvHD [32]. However, despite the promising results reported by de Lima et al., the randomized phase III trial conducted by Oran et al. failed to demonstrate a statistically significant advantage of AZA [33]. Indeed, two recent meta-analyses based on retrospective studies using HMAs as maintenance therapy demonstrated a superiority in OS and EFS compared to the observational groups [34]. From the same meta-analysis, it emerged that AZA and DEC seem more effective in high-risk MDS and AML enhancing the GVL effect [35]. Based on the above results of the QUAZAR trial, the oral formulation of AZA (CC-486) was investigated in the ongoing phase III AMADEUS (NCT04173533) trial in the post-HSCT setting [36]. Conversely, in the setting of MRD positive post-HSCT, AZA has been administered at higher dosages, ranging from 75 mg/m^2^ for 7 days to 100 mg/m^2^ for 5 days, both in retrospective and prospective studies. The RELAZA prospective trials conducted by Platzbecker and colleagues enrolled 20 patients allografted for MDS and high-risk AML in CR with a decrease in CD34+ donor chimerism. They received four cycles (75 mg/m^2^ day for 7 days) of AZA, demonstrating that pre-emptive AZA has an acceptable safety profile and was able to prevent or delay hematologic relapse [37]. The same group evaluated AZA as a pre-emptive strategy in an MRD-driven phase II trial. A group of these patients underwent HSCT and were monitored with chimerism analysis. Overall, the results showed that an early intervention with AZA in MRD-positive patients can prevent or substantially delay hematologic relapse, allowing patients to recover from previous toxicities, becoming eligible for subsequent treatment [38]. Woo et al. conducted a prospective trial on 39 patients with early (<100 days) relapse (hematological or MRD) after-HSCT [39]. They found that the intensity of pre-HSCT chemotherapy and the burden of disease at the time of relapse were factors determining the response to AZA. Overall, AZA as maintenance treatment is a valid option considering the limited toxicity. However, further larger and randomized clinical trials are mandatory to confirm and approve the drug. DEC was demonstrated to be potentially useful as maintenance therapy post-HSCT. With the use of DEC at a dosage of 10 mg/m^2^, the 2-year OS and CIR were 56% and 28%, respectively. Next, a phase II multicenter randomized trial investigated DEC as maintenance in 204 high-risk patients who were MRD negative post allo-HSCT [40]. The randomization was planned to received G-CSF in combination with low-dose DEC (LDEC) for 5 days (G-DEC) or no intervention. The CIR was lower in the G-DEC group and demonstrated a lower CIR compared with the no-intervention group (15% versus 38%, respectively). Indeed, LDEC at a dosage of 15 mg/m^2^ for 3 days was explored in combination with VEN (200 mg on day 1–21) in the maintenance phase post-allo-HSCT. Wei et al. reported the results of 20 high-risk MDS/AML patients. The CIR after LDEC + VEN was 15.3%, and the 2-year NRM was 6.1% [41]. Furthermore, LDEC with VEN did not increase the GVHD.

### HMAs in Combination with Venetoclax

The anti-apoptotic BCL2 inhibitor venetoclax (VEN) in combination with HMA or with a low dose of cytarabine has shown unprecedented efficacy in AML, and it is currently approved by the FDA and EMA for the treatment of first-line unfit AML patients [42]. The efficacy of this combo was also demonstrated in the setting of R/R AML [43]. In the post-HSCT, the major experience is in combination with HMA in the setting of frank hematological relapse (see below), while in the maintenance phase, a single experience of VEN as a single agent was reported by Kent et al. in 49 patients treated with VEN 400 mg daily [44]. The 1-year RFS and OS were 67% and 70%, respectively. The drug was also found to be safe and well tolerated in terms of GVHD reduction. The most attractive combination for the maintenance phase post-HSCT is VEN-AZA. Oran et al. reported the preliminary results of a phase II clinical trial in which VEN at 100 mg/day and AZA at 32 mg/m^2^/day 1–5 were administered as the maintenance phase. A total of 30 patients were enrolled (27 AML and 3 ALL) with a median follow-up of 8.67 months (95%, CI 7.23 to 13.6). The median RFS and OS were not reached. The estimates of RFS and OS at 1 year were 69.2% (95% CI, 52.1% to 91.8%) and 90.2% (95%CI, 78% to 100%), respectively, after the initiation of VEN + AZA maintenance. Due to hematological toxicity, the dosage of AZA was reduced to 50 mg. Despite the greater toxicity, the combination of VEN-AZA was effective; however, the data need to be confirmed in a longer follow-up. The current phase III VIALE-T clinical study is in fact studying VEN-AZA in post-HSCT maintenance, using a 28-day administration for 6 cycles followed by VEN monotherapy for up to 24 cycles. The combination of DEC at 10 mg/m^2^ for 3 days + VEN 200 mg was tested by Parks et al. in 26 patients with high-risk disease as maintenance post-HSCT. The 1-year non-relapse mortality and OS were 11% and 84%, respectively [45]. Moreover, in the study of Wei et al., DEC at 15 mg/m^2^ plus VEN 200 mg on day 1–21 up to 10 cycles post-HSCT demonstrated efficacy with a 1-year relapse incidence of 15.3% and a 2-year OS of 85.2% [41].

## 6. Donor Lymphocyte Infusion (DLI)

The immunotherapeutic rationale of HSCT is based on the GvL effect through the alloreactivity of donor T cells against malignant cells. This means that the balance between the therapeutic effect of GvL and the toxic effect of GvHD must be carefully considered. For more than 30 years, DLIs have represented an attractive therapeutic option to reduce the risk of relapse post-HSCT, enhancing the immunological surveillance [46,47]. The donor lymphocytes commonly used in clinical practice are unmanipulated. DLI can be collected and cryopreserved during the previous transplant or after a second apheresis. The composition is mostly represented by T cell subsets (80–90%), NK-cells (5–20%), and B-cells (5%). Among T cells, the most represented subset is αβ followed by γδ [47]. Nowadays, due to the lack of prospective clinical trials and the presence of retrospective heterogeneous results, guidelines are not available; however, the EBMT group drafted recommendations for clinical application of DLI based on expert consensus. The largest body of evidence derived from retrospective series; however, the results are heterogeneous based on disease status and the dosage and timing of DLI administration. Taken together, OS and the incidence of relapse ranged from 25 to 80% and 20 to 43%, respectively. The major limitation of DLI is represented by the onset of chronic GvHD, occurring between 30 and 55% of cases. The EBMT group published the results of a retrospective study demonstrating an advantage in OS for DLI treatment compared with those not receiving it (OS 21% versus 9%) [48,49]. The clinical setting for the application of DLI are the hematological relapse, the pre-emptive—when MRD is positive and/or the chimerism is mixed—and the prophylactic or maintenance approach. Because frank hematological relapse has proven to nullify the DLI effect, Dominietto et al. investigated the protective role of DLI infusion in the MRD-positive setting [50]. The study showed that MRD-positive patients treated with DLIs experienced a lower relapse rate compared those who did not receive them. Subsequently, two prospective trials demonstrated the advantage of pre-emptive DLI in reducing relapse rates [49,51]. A further step forward was the administration of DLI as maintenance post-HSCT. Schmid et al. reported a matched-pair analysis on 89 patients and 89 control cases showing that the DLI group presented a superior OS, mostly in a very high-risk subgroup [52]. Indeed, the experience of Legrand showed that cases treated with DLI in maintenance had a 2-year OS rate of 75% and a relapse rate of 22% [53]. Similarly, Jedickova et al. reported a lower relapse rate (22% vs. 53%) and a better OS (67% vs. 31%) for patients who received DLI as maintenance [54]. Couchois et al. investigated DLI application in 36 patients who performed haploidentical-HSCT using post-transplant cyclophosphamide (PTCy) as GvHD prophylaxis [55]. Twenty-five AML/MDS patients showed a 1-year OS and progression-free survival (PFS) of 83% and 76%, respectively. The incidence of GvHD was 33%, similar to the incidence of transplants with different types of donors. Finally, Santoro et al., in a multicenter study, compared the DLI used in prophylaxis, preemptive, and frank relapse and demonstrated that the prophylactic strategy was associated with a better OS. However, the incidence of cGvHD was higher [56]. In order to clarify the incidence and severity of GvHD with the use of DLI in maintenance, a prospective randomized phase III study is ongoing (ELIT-AML trial NCT0067442-Early prophylactic donor Lymphocytes Infusion after allogeneic Transplantation for AML). In the absence of definitive studies, the EBMT has in the meantime published the following recommendations in the field of post-HSCT maintenance (Table 2):

1. The indication for DLI is for patients with high-risk relapse, according to one of the following three factors:
  - high-risk biological features;
  - transplantation in refractory or advanced stage;
  - ex vivo lymphocyte depletion as GvHD prophylaxis.
2. Prophylactic DLI is indicated in transplants using a non-myeloablative conditioning regimen or in the absence of molecular targets, independently of the conditioning regimen;
3. The first infusion should be administered after immunosuppression for >30 days; it is mandatory that there be an absence of active GvHD and infections;
4. The interval from HSCT to the first DLI was reported to be 4–6 months; however, an early application is recommended in high-risk cases;
5. The dose of infusions would increase 0.5–1 log and the interval would be 4–6 weeks;
6. MRD, chimerism monitoring, and the onset of GvHD would drive the DLI frequencies; however, 1–3 doses are recommended.

In the table, the dosage of DLI based on the different types of donors is reported.

## 7. Conclusions

The maintenance therapy in AML is a complex challenge. This complexity arises from several factors, including the lack of robust treatment efficacy, which is partly due to the scarcity of large-scale, randomized prospective clinical trials. The limited availability of approved drugs further complicates the landscape, leaving clinicians with few standardized options. Additionally, the clinical and psychological complexity of patients who undergo HSCT adds another layer of difficulty, as these individuals often face significant physical and emotional burdens that can impact treatment adherence and outcomes. Given these challenges, the choice of a maintenance strategy must be carefully tailored to each patient. As the field advances, more research is urgently needed to establish evidence-based guidelines to drive clinical practice and finally improve patient outcome.

## Figures and Tables

**Table 1:** Main post-transplant maintenance trials in AML.

Study	Phase	Agents	Schedule	Results	Side Effects
NCT02474290	III	Sorafenib vs. placebo	Sorafenib 400 mg bid for 6 months	Improved 2-year RFS (78.9% vs. 56.6%) and OS (82.1% vs. 68.0%) with sorafenib	myelosuppression
NCT02997202	III	Gilteritinib vs. placebo	Gilteritinib 120 mg for 24 months	Improved RFS in MRD+ (HR = 0.515, 95% CI: 0.316, 0.838, *p* = 0.0065) with gilteritinib	Important myelosuppression
NCT03564821	I	Ivosidenib	Ivosidenib 500 mg and 250 mg	2-year relapse = 19% 2-year PFS = 81% 2-year OS = 88%	No increased GvHD
NCT03515512	I	Enasidenib	Enasidenib 100 mg and 50 mg	Relapse = 16% 2-year PFS = 69% 2-year OS = 74%	No increased GvHD
NCT03931291	II	APR246 + AZA in		1-year RFS = 59.9% 1-year OS = 78.8%	No increased GvHD
NCT00887068	III	AZA vs. placebo	AZA 32 mg/m^2^ for 5 days	RFS: 2.07 years (AZA) vs. 1.28 years (placebo), *p* = 0.43	myelosuppression
ChiCTR1900025374	II	DEC/VEN	DEC 15 mg/m^2^ for 3 days Ven 200 mg days 1–21	1-year relapse = 15.3% 1-year OS = 85.2%	Reversible myelosuppression
NCT03613532	I	AZA/VEN	AZA 36 mg/m^2^ for 5 days Ven 400 mg days 1–14	2-year relapse = 41% 2-year OS = 67%	Reversible myelosuppression

**Table 2:** Recommended doses according to different HSCT. Adapted from Pagliuca S et al [46].

	DLI Dose for Matched Related Donor	DLI Dose for Matched Unrelated Donor	DLI Dose for Mismatched Unrelated Donor or Haploidentical Donor
3 months	1 × 10^5^ cells/kg × 1–3 doses	1 × 10^5^ cells/kg 1–3 doses	1 × 10^5^ cells/kg 1–3 doses
6 months	1 × 10^6^ cells/kg	1 × 10^6^ cells/kg	5 × 10^5^ cells/kg

## References

[1] Shimony S., Stahl M., Stone R.M. (2023). Acute myeloid leukemia: 2023 update on diagnosis, risk-stratification, and management. Am. J. Hematol..

[2] Döhner H., Wei A.H., Appelbaum F.R., Craddock C., DiNardo C.D., Dombret H., Ebert B.L., Fenaux P., Godley L.A., Hasserjian R.P. (2022). Diagnosis and management of AML in adults: 2022 recommendations from an international expert panel on behalf of the ELN. Blood.

[3] Snowden J.A., Sánchez-Ortega I., Corbacioglu S., Basak G.W., Chabannon C., de la Camara R., Dolstra H., Duarte R.F., Glass B., Greco R. (2022). Indications for haematopoietic cell transplantation for haematological diseases, solid tumours and immune disorders: Current practice in Europe, 2022. Bone Marrow Transplant..

[4] Dholaria B., Savani B.N., Hamilton B.K., Oran B., Liu H.D., Tallman M.S., Ciurea S.O., Holtzman N.G., Ii G.L.P., Devine S.M. (2021). Hematopoietic Cell Transplantation in the Treatment of Newly Diagnosed Adult Acute Myeloid Leukemia: An Evidence-Based Review from the American Society of Transplantation and Cellular Therapy. Transplant. Cell. Ther..

[5] Molica M., Breccia M., Foa R., Jabbour E., Kadia T.M. (2019). Maintenance therapy in AML: The past, the present and the future. Am. J. Hematol..

[6] Wei A.H., Döhner H., Pocock C., Montesinos P., Afanasyev B., Dombret H., Ravandi F., Sayar H., Jang J.-H., Porkka K. (2020). Oral Azacitidine Maintenance Therapy for Acute Myeloid Leukemia in First Remission. N. Engl. J. Med..

[7] DeFilipp Z., Chen Y.B. (2023). How I treat with maintenance therapy after allogeneic HCT. Blood.

[8] Al-Shaibani E., Novitzky-Basso I., Mattsson J., Kim D.D.H. (2023). Post-transplant maintenance therapy in acute myeloid leukemia after allogeneic hematopoietic stem cell transplantation harmonizing multiple therapeutic modalities including targeted therapy, immunotherapy and cellular therapy. Int. J. Hematol..

[9] Papaemmanuil E., Gerstung M., Bullinger L., Gaidzik V.I., Paschka P., Roberts N.D., Potter N.E., Heuser M., Thol F., Bolli N. (2016). Genomic classification and prognosis in acute myeloid leukemia. N. Engl. J. Med..

[10] Meshinchi S., Stirewalt D.L., Alonzo T.A., Boggon T.J., Gerbing R.B., Rocnik J.L., Lange B.J., Gilliland D.G., Radich J.P. (2008). Structural and numerical variation of FLT3/ITD in pediatric AML. Blood.

[11] Santos F.P., Jones D., Qiao W., Cortes J.E., Ravandi F., Estey E.E., Verma D., Kantarjian H., Borthakur G. (2011). Prognostic value of FLT3 mutations among different cytogenetic subgroups in acute myeloid leukemia. Cancer.

[12] Takahashi S. (2011). Downstream molecular pathways of FLT3 in the pathogenesis of acute myeloid leukemia: Biology and therapeutic implications. J. Hematol. Oncol..

[13] Choudhary C., Schwable J., Brandts C., Tickenbrock L., Sargin B., Kindler T., Fischer T., Berdel W.E., Muller-Tidow C., Serve H. (2005). AML-Associated Flt3 kinase domain mutations show signal transduction differences compared with Flt3 ITD mutations. Blood.

[14] Bazarbachi A., Bug G., Baron F., Brissot E., Ciceri F., Dalle I.A., Döhner H., Esteve J., Floisand Y., Giebel S. (2020). Clinical practice recommendationon hematopoietic stem cell transplantation for acute myeloid leukemia patients with FLT3-internal tandem duplication: A position statement from the Acute Leukemia Working Party of the European Society for Blood and Marrow Transplantation. Haematologica.

[15] Antar A.I., Otrock Z.K., Jabbour E., Mohty M., Bazarbachi A. (2020). FLT3 inhibitors in acute myeloid leukemia: Ten frequently asked questions. Leukemia.

[16] Mathew N.R., Baumgartner F., Braun L., O’Sullivan D., Thomas S., Waterhouse M., AMüller T., Hanke K., Taromi S., Apostolova P. (2018). Sorafenib Promotes Graft-Versus-Leukemia Activity in Mice and Humans Through IL-15 Production in FLT3-ITD-Mutant Leukemia Cells. Nat. Med..

[17] Burchert A., Bug G., Fritz L.V., Finke J., Stelljes M., Röllig C., Wollmer E., Wäsch R., Bornhäuser M., Berg T. (2020). Sorafenib Maintenance After Allogeneic Hematopoietic Stem Cell Transplantation for Acute Myeloid Leukemia With FLT3-Internal Tandem Duplication Mutation (SORMAIN). J. Clin. Oncol..

[18] Xuan L., Wang Y., Yang K., Shao R., Huang F., Fan Z., Chi P., Xu Y., Xu N., Deng L. (2023). Sorafenib maintenance after allogeneic haemopoietic stem-cell transplantation in patients with FLT3-ITD acute myeloid leukaemia: Long-term follow-up of an open-label, multicentre, randomised, phase 3 trial. Lancet Haematol..

[19] Perl A.E., Larson R.A., Podoltsev N.A., Strickland S., Wang E.S., Atallah E., Schiller G.J., Martinelli G., Neubauer A., Sierra J. (2022). Follow-up of patients with R/R FLT3-mutation-positive AML treated with gilteritinib in the phase 3 ADMIRAL trial. Blood.

[20] Levis M.J., Hamadani M., Logan B., Jones R.J., Singh A.K., Litzow M., Wingard J.R., Papadopoulos E.B., Perl A.E., Soiffer R.J. (2024). Gilteritinib as Post-Transplant Maintenance for AML With Internal Tandem Duplication Mutation of *FLT3*. J. Clin. Oncol..

[21] Stone R.M., Mandrekar S.J., Sanford B.L., Laumann K., Geyer S., Bloomfield C.D., Thiede C., Prior T.W., Döhner K., Marcucci G. (2017). Midostaurin plus chemotherapy for acute myeloid leukemia with a FLT3 mutation. N. Engl. J. Med..

[22] Erba H.P., Montesinos P., Kim H.-J., Patkowska E., Vrhovac R., Žák P., Wang P.-N., Mitov T., Hanyok J., Kamel Y.M. (2023). Quizartinib plus chemotherapy in newly diagnosed patients with *FLT3*-internal-tandem-duplication-positive acute myeloid leukaemia (QuANTUM-First): A randomised, double-blind, placebo-controlled, phase 3 trial. Lancet.

[23] DiNardo C.D., Ravandi F., Agresta S., Konopleva M., Takahashi K., Kadia T., Routbort M., Patel K.P., Mark B., Pierce S. (2015). Characteristics, clinical outcome, and prognostic significance of IDH mutations in AML.. Am. J. Hematol..

[24] Chen E.C., Li S., Eisfeld A.-K., Luskin M.R., Mims A., Jones D., Antin J.H., Cutler C.S., Koreth J., Ho V.T. (2021). Outcomes for patients with IDH-mutated acute myeloid leukemia undergoing allogeneic hematopoietic cell transplantation. Transplant. Cell. Ther..

[25] Salhotra A., Afkhami M., Yang D., Mokhtari S., Telatar M., Gu D., Pillai R.K., Weisenburger D.D., Murata-Collins J., Weigel D. (2019). Allogeneic hematopoietic cell transplantation outcomes in patients carrying isocitrate dehydrogenase mutations. Clin. Lymphoma Myeloma Leuk..

[26] Mishra A., Tamari R., DeZern A.E., Byrne M.T., Gooptu M., Chen Y.-B., Deeg H.J., Sallman D., Gallacher P., Wennborg A. (2022). Eprenetapopt Plus Azacitidine After Allogeneic Hematopoietic Stem-Cell Transplantation for TP53-Mutant Acute Myeloid Leukemia and Myelodysplastic Syndromes. J. Clin. Oncol..

[27] Byrne M.T., Kurian T.J., Patel A.D., Tamari R., Hong S., Abdelhakim H., Klein V., Rojas P., Madhavan R., Kent A. (2021). Non-relapse mortality in TP53-mutated MDS/AML—A multi-center collaborative study. Blood.

[28] Lindsley R.C., Saber W., Mar B.G., Redd R., Wang T., Haagenson M.D., Grauman P.V., Hu Z.H., Spellman S.R., Lee S.J. (2017). Prognostic mutations in myelodysplastic syndrome after stem-cell transplantation. N. Engl. J. Med..

[29] Manobianco S.A., Rakiewicz T., Wilde L., Palmisiano N.D. (2022). Novel Mechanisms for Post-Transplant Maintenance Therapy in Acute Myeloid Leukemia. Front. Oncol..

[30] de Lima M., Giralt S., Thall P.F., de Padua Silva L., Jones R.B., Komanduri K., Braun T.M., Nguyen H.Q., Champlin R., Garcia-Manero G. (2010). Maintenance therapy with low-dose azacitidine after allogeneic hematopoietic stem cell transplantation for recurrent acute myelogenous leukemia or myelodysplastic syndrome: A dose and schedule finding study. Cancer.

[31] Goodyear O.C., Dennis M., Jilani N.Y., Loke J., Siddique S., Ryan G., Nunnick J., Khanum R., Raghavan M., Cook M. (2012). Azacitidine augments expansion of regulatory T cells after allogeneic stem cell transplantation in patients with acute myeloid leukemia (AML). Blood.

[32] Schroeder T., Fröbel J., Cadeddu R.-P., Czibere A., Dienst A., Platzbecker U., Bug G., Uharek L., Fenk R., Germing U. (2013). Salvage therapy with azacitidine increases regulatory T cells in peripheral blood of patients with AML or MDS and early relapse after allogeneic blood stem cell transplantation. Leukemia.

[33] Oran B., de Lima M., Garcia-Manero G., Thall P.F., Lin R., Popat U., Alousi A.M., Hosing C., Giralt S., Rondon G. (2020). A phase 3 randomized study of 5-azacitidine maintenance vs observation after transplant in high-risk AML and MDS patients. Blood Adv..

[34] Liu W., Zhou Z., Chen L., Wang X. (2021). Comparison of Azacitidine and Decitabine in Myelodysplastic Syndromes and Acute Myeloid Leukemia: A Network Meta-analysis. Clin. Lymphoma Myeloma Leuk..

[35] Kungwankiattichai S., Ponvilawan B., Roy C., Tunsing P., Kuchenbauer F., Owattanapanich W. (2022). Maintenance With Hypomethylating Agents After Allogeneic Stem Cell Transplantation in Acute Myeloid Leukemia and Myelodysplastic Syndrome: A Systematic Review and Meta-Analysis. Front. Med..

[36] de Lima M., Oran B., Champlin R.E., Papadopoulos E.B., Giralt S.A., Scott B.L., William B.M., Hetzer J., Laille E., Hubbell B. (2016). CC-486 (oral azacitidine) maintenance therapy is well tolerated after allogeneic hematopoietic stem cell transplantation (alloHSCT) in patients with myelodysplastic syndromes (MDS) or acute myeloid leukemia (AML). Biol. Blood Marrow Transplant..

[37] Platzbecker U., Wermke M., Radke J., Oelschlaegel U., Seltmann F., Kiani A., Klut I.M., Knoth H., Röllig C., Schetelig J. (2012). Azacitidine for treatment of imminent relapse in MDS or AML patients after allogeneic HSCT: Results of the RELAZA trial. Leukemia.

[38] Platzbecker U., Middeke J.M., Sockel K., Herbst R., Fransecky L.R., Wolf D., Martin S., Krämer A., Noppeney R., Novotny J. (2019). Azacitidine for Pre-Emptive Treatment of Measurable-Residual Disease in MDS/AML Patients at High Risk of Hematological Relapse: Results of the Second Cohort of the RELAZA2 Trial. Blood.

[39] Woo J., Deeg H.J., Storer B., Yeung C., Fang M., Mielcarek M., Scott B.L. (2017). Factors Determining Responses to Azacitidine in Patients with Myelodysplastic Syndromes and Acute Myeloid Leukemia with Early Post-Transplantation Relapse: A Prospective Trial. Biol. Blood Marrow Transplant..

[40] Gao L., Zhang Y., Wang S., Kong P., Su Y., Hu J., Jiang M., Bai H., Lang T., Wang J. (2020). Effect of rhG-CSF Combined with Decitabine Prophylaxis on Relapse of Patients with High-Risk MRD-Negative AML after HSCT: An Open-Label, Multicenter, Randomized Controlled Trial. J. Clin. Oncol..

[41] Wei Y., Xiong X., Li X., Lu W., He X., Jin X., Sun R., Lyu H., Yuan T., Sun T. (2021). Low-dose decitabine plus venetoclax is safe and effective as post-transplant maintenance therapy for high-risk acute myeloid leukemia and myelodysplastic syndrome. Cancer Sci..

[42] Dinardo C.D., Pratz K., Pullarkat V., Jonas B.A., Arellano M., Becker P.S., Frankfurt O., Konopleva M., Wei A.H., Kantarjian H.M. (2019). Venetoclax combined with decitabine or azacitidine in treatment-naive, elderly patients with acute myeloid leukemia. Blood.

[43] Wei A.H., Strickland S.A., Hou J.-Z., Fiedler W., Lin T., Walter R.B., Enjeti A., Tiong I.S., Savona M., Lee S. (2019). Venetoclax Combined With Low-Dose Cytarabine for Previously Untreated Patients With Acute Myeloid Leukemia: Results From a Phase Ib/II Study. J. Clin. Oncol..

[44] Kent A., Schwartz M., McMahon C., Amaya M., Smith C.A., Tobin J., Marciano K., Rezac R., Bosma G., Pollyea D.A. (2023). Venetoclax is safe and tolerable as post-transplant maintenance therapy for AML patients at high risk for relapse. Bone Marrow Transpl..

[45] Parks K., Diebold K., Salzman D., Di Stasi A., Al-Kadhimi Z., Espinoza-Gutarra M., Bhatia R., Jamy O. (2024). Low-dose decitabine plus venetoclax as post-transplant maintenance for high-risk myeloid malignancies. eJHaem.

[46] Pagliuca S., Schmid C., Santoro N., Simonetta F., Battipaglia G., Guillaume T., Greco R., Onida F., Sánchez-Ortega I., Yakoub-Agha I. (2024). Donor lymphocyte infusion after allogeneic haematopoietic cell transplantation for haematological malignancies: Basic considerations and best practice recommendations from the EBMT. Lancet Haematol..

[47] Schmid C., Kuball J., Bug G. (2021). Defining the Role of Donor Lymphocyte Infusion in High-Risk Hematologic Malignancies. J. Clin. Oncol..

[48] Frederik F.J.H., Schmid C., Kolb H.J., Locatelli F., Kuball J. (2019). Delayed transfer of immune cells or the art of donor lymphocyte infusion. The EBMT Handbook: Hematopoietic Stem Cell Transplantation and Cellular Therapies.

[49] Levine J.E., Braun T., Penza S.L., Beatty P., Cornetta K., Martino R., Drobyski W.R., Barrett A.J., Porter D.L., Giralt S. (2002). Prospective trial of chemotherapy and donor leukocyte infusions for relapse of advanced myeloid malignancies after allogeneic stem-cell transplantation. J. Clin. Oncol..

[50] Dominietto A., Pozzi S., Miglino M., Albarracin F., Piaggio G., Bertolotti F., Grasso R., Zupo S., Raiola A.M., Gobbi M. (2007). Donor lymphocyte infusions for the treatment of minimal residual disease in acute leukemia. Blood.

[51] Schmid C., Labopin M., Schaap N., Veelken H., Brecht A., Stadler M., Finke J., Baron F., Collin M., Bug G. (2022). Long-term results and GvHD after prophylactic and preemptive donor lymphocyte infusion after allogeneic stem cell transplantation for acute leukemia. Bone Marrow Transplant..

[52] Schmid C., Labopin M., Schaap N., Veelken H., Schleuning M., Stadler M., Finke J., Hurst E., Baron F., Ringden O. (2019). Prophylactic donor lymphocyte infusion after allogeneic stem cell transplantation in acute leukaemia—A matched pair analysis by the Acute Leukaemia Working Party of EBMT. Br. J. Haematol..

[53] Legrand F., Le Floch A.-C., Granata A., Fürst S., Faucher C., Lemarie C., Harbi S., Bramanti S., Calmels B., El-Cheikh J. (2017). Prophylactic donor lymphocyte infusion after allogeneic stem cell transplantation for high-risk AML.. Bone Marrow Transplant..

[54] Jedlickova Z., Schmid C., Koenecke C., Hertenstein B., Baurmann H., Schwerdtfeger R., Tischer J., Kolb H.J., Schleuning M. (2016). Long-term results of adjuvant donor lymphocyte transfusion in AML after allogeneic stem cell transplantation. Bone Marrow Transplant..

[55] Cauchois R., Castagna L., Pagliardini T., Harbi S., Calmels B., Bramanti S., Granata A., Lemarie C., Maisano V., Legrand F. (2019). Prophylactic donor lymphocyte infusions after haploidentical haematopoietic stem cell transplantation for high risk haematological malignancies: A retrospective bicentric analysis of serial infusions of increasing doses of CD3^+^ cells. Br. J. Haematol..

[56] Santoro N., Mooyaart J.E., Devillier R., Koc Y., Vydra J., Castagna L., Gülbas Z., Martin J.D., Araujo M.C., Kulagin A. (2023). Donor lymphocyte infusions after haploidentical stem cell transplantation with PTCY: A study on behalf of the EBMT cellular therapy & immunobiology working party. Bone Marrow Transplant..

